# Promoter-Specific Expression and Imprint Status of Marsupial *IGF2*

**DOI:** 10.1371/journal.pone.0041690

**Published:** 2012-07-25

**Authors:** Jessica M. Stringer, Shunsuke Suzuki, Andrew J. Pask, Geoff Shaw, Marilyn B. Renfree

**Affiliations:** 1 ARC Centre of Excellence in Kangaroo Genomics, The University of Melbourne, Victoria, Australia; 2 Department of Zoology, The University of Melbourne, Victoria, Australia; 3 Epigenomics Division, Frontier Agriscience and Technology Center, Faculty of Agriculture, Shinshu University, Nagano, Japan; 4 Department of Molecular and Cellular Biology, The University of Connecticut, Storrs, Connecticut, United States of America; The Walter and Eliza Hall of Medical Research, Australia

## Abstract

In mice and humans, *IGF2* has multiple promoters to maintain its complex tissue- and developmental stage-specific imprinting and expression. *IGF2* is also imprinted in marsupials, but little is known about its promoter region. In this study, three *IGF2* transcripts were isolated from placental and liver samples of the tammar wallaby, *Macropus eugenii*. Each transcript contained a unique 5' untranslated region, orthologous to the non-coding exons derived from promoters P1–P3 in the human and mouse *IGF2* locus. The expression of tammar *IGF2* was predominantly from the P2 promoter, similar to humans. Expression of *IGF2* was higher in pouch young than in the adult and imprinting was highly tissue and developmental-stage specific. Interestingly, while *IGF2* was expressed throughout the placenta, imprinting seemed to be restricted to the vascular, trilaminar region. In addition, *IGF2* was monoallelically expressed in the adult mammary gland while in the liver it switched from monoalleleic expression in the pouch young to biallelic in the adult. These data suggest a complex mode of *IGF2* regulation in marsupials as seen in eutherian mammals. The conservation of the *IGF2* promoters suggests they originated before the divergence of marsupials and eutherians, and have been selectively maintained for at least 160 million years.

## Introduction

Insulin-like growth factor 2 *(IGF2)* is an important regulator of growth, metabolism and differentiation and was the first imprinted gene identified in eutherians and in marsupials [1], [2]. The coding region of *IGF2* is highly conserved across all mammals, but the non-coding exons are variable. Human *IGF2* has five promoters (huP1 and P0–P3) and transcription start sites (TSS) each adjoining distinct 5′ non-coding exons, while rodent *IGF2* has only 4 (P0–P3) [3]–[12]. Preliminary investigations in the South American marsupial, *Monodelphis domestica*, detected a single product from total neonatal RNA using 5′ RACE and identified only one 5′ non-coding exon [13]. Thus, opossum neonates appear to produce only one *IGF2* isotype from a single promoter.

Human *IGF2* is imprinted in the fetal liver and is paternally expressed from P1, P2 and P3 (orthologous to mouse P1–P3 respectively) with dominant expression from the P2 promoter in the majority of tissues tested [14]. In the adult liver *IGF2* has continued expression from P1 and P3 in addition to biallelic expression from huP1 (previously reported as P1) [14], [15]. There is no functional mouse homologue for huP1 which is located 20 kb upstream of P1–P3. In the human, reciprocal activation patterns of P2 and huP1 before and after birth respectively have been correlated to developmental-specific methylation at the two promoters [16].

In the mouse P0-derived transcripts are only expressed in the labyrinthine layer of the placenta, whereas transcripts from P1–P3 are found throughout the developing embryo and placenta with dominant expression from the P3 promoter [17]–[21]. Mouse *Igf2* is paternally expressed in the embryo, but biallelically expressed in the choroid plexus and leptomeniges, which are the only major sites of *Igf2* gene expression in the adult rodents [1], [22]–[24]. The human *IGF2-P0* transcript is paternally expressed at high levels in fetal skeletal muscle, in the term placenta and at low levels in all adult tissues except the brain [12].

The mouse *Igf2* domain has 3 differentially methylated regions (DMRs), DMR0 surrounding the area that codes for the placental specific promoter P0, DMR1 located 5′ of P1 and DMR2 located at the second coding exon [12], [25]. In the mouse, both DMR 1 and 2 are paternally hypermethylated. DMR1 is a mesodermal silencer. Therefore, deletion of this region results in biallelic expression of *IGF2* in mesoderm-derived tissues (heart, kidney and lung) without disrupting *H19* imprinting, whilst maintaining *IGF2* imprinting in the endoderm-derived tissues (liver and placenta) [26]. Deletion of DMR2 has no effect on imprinting but reduces transcriptional activation of *IGF2*, therefore DMR2 is an activation site [27]. Humans lack DMR1 [18], but DMR2 is present and paternally hypermethylated [28]. In addition methylation of this region varies within tissues of patients with Beckwith-Wiedemann syndrome, suggesting a similar function to that seen in the mouse [28]. In humans, the promoter-specific methylation of P1–P3 spreads from the silenced maternal allele to the transcriptionally active paternal allele [29]. The increase in methylation on the paternal allele with age potentially regulates the level of expression from each promoter. This promoter specific methylation pattern is not seen in the mouse [30].

**Figure 1:**
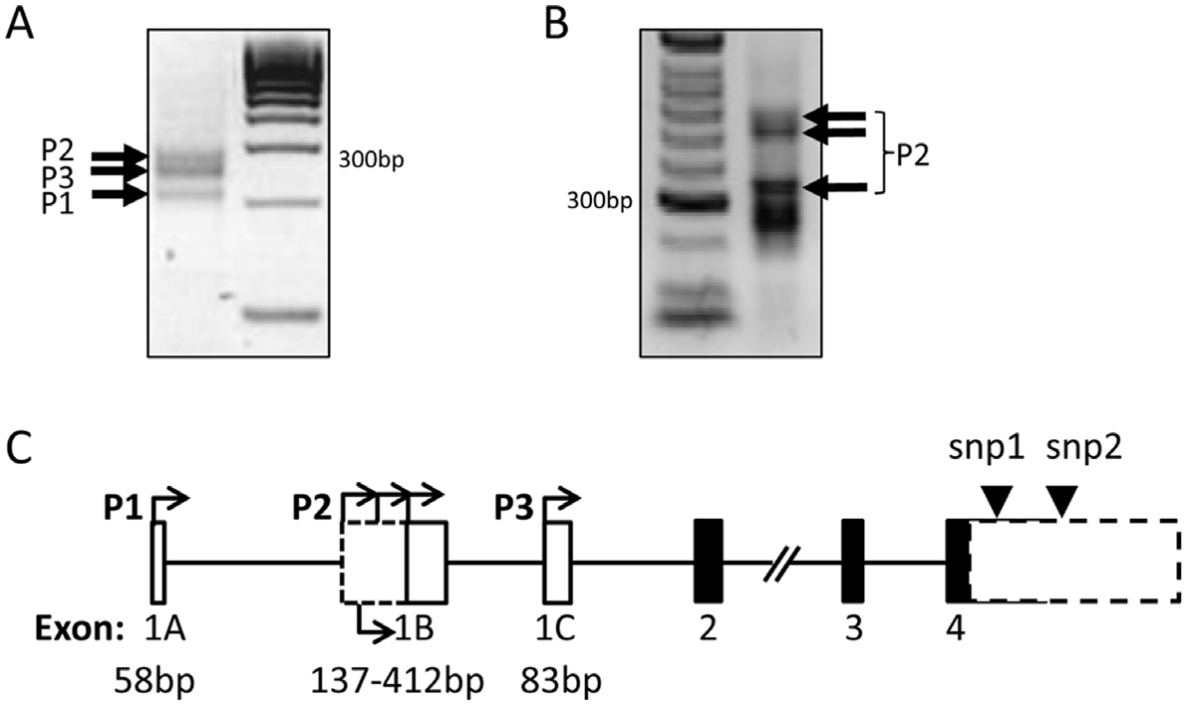
Identifying tammar *IGF2* transcription start sites. 5′-RACE amplification was performed on (A) liver and (B) placenta RNA samples. (A) Three bands first identified in the liver (black arrows) were visible in both tissue types. (B) Additional exon 1B transcription start sites (TSS) were identified in the placenta. (C) Aligning the cloned products to the gDNA sequence identified the location of each of the non-coding exons 1A, 1B and 1C (white boxes) in relation to the coding exons 2, 3 and 4 (black boxes) followed by an untranslated region of unknown length (dashed box). TSSs are indicated by turned arrows. Two single nucleotide polymorphisms (black triangles) identified in the untranslated region were used for imprint analysis (snp1 and snp2). Diagram is not to scale.

The presence of a potential functional equivalent of mouse DMR2 [28] and DMR0 [12] and the absence of DMR1 [18] in the human, coupled with the occurrence of parental allele-specific methylation in the promoter regions of the human, but not the mouse [29], [30], suggests a divergence of *IGF2* control mechanisms amongst the eutherian mammals. Marsupials diverged from the eutherian mammals around 160 million years ago [31], yet *IGF2* is highly conserved and imprinted. However, very little is known about the regulation of this gene in marsupials. Most marsupials have a short pregnancy supported by a much less invasive placenta than typically seen in eutherian mammals, and all give birth to a highly altricial neonate [32]. Therefore, conserved promoter elements between marsupials and eutherians are expected to be critical in the function and regulation of the *IGF2* gene.

**Figure 2:**
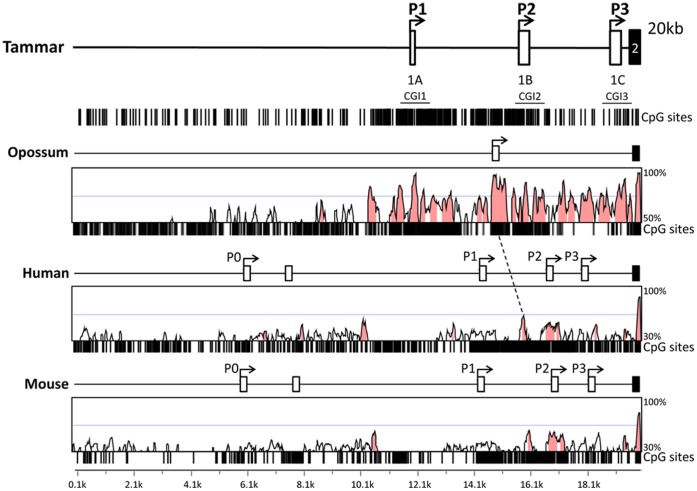
Genomic structure of tammar IGF2 including 3 TSS and comparison with mouse, human and opossum genomes. Opossum (clone XX-223O16 ch5∶102112-82113), human (ch11∶2156597-2176597) and mouse (ch7∶149841559-149861559), *IGF2* base genomes were aligned to tammar *IGF2* (clone MEKBa-346C2∶62540-82539). VISTA pairwise alignments using a 100 bp sliding window was performed between each species and tammar. Pink peaks represent areas of conservation of 70% over a minimum of 100 bp between tammar and opossum and 55% over a minimum of 50 bp between tammar and human or mouse. A dotted line indicates a region with 70% conservation between P1 and P2 in both marsupials and eutherians. A schematic of each *IGF2* gene is located above the VISTA plot to show relative location of the non-coding exons (open box), including the non-coding exons transcribed with the P0 TSS [12], and coding exons (black box). Location of CpG sites are represented by vertical black bars below the VISTA plot.

*IGF2* is imprinted in the whole bodies of opossum neonates [2], [13]. In the tammar wallaby, *IGF2* shows complete imprinting in the fetal liver, but has paternally biased expression in the placenta and brain and is biallelically expressed adult liver [33]–[35]. There is a 95 base pair non-coding exon upstream of the first protein coding exon of *IGF2* in the neonatal opossum [13]. In monotemes only a single transcript has been characterized with no non-coding exons [36]. In the tammar, there are several different 5′ sequences produced from the *IGF2* gene that may account for the biased expression observed in the head and the placenta [34]. However, as yet no one has identified the transcription start sites (TSSs) of tammar *IGF2*. This study investigates the evolution of the *IGF2* regulatory regions in a marsupial, the tammar wallaby *Macropus eugenii,* and identified 3 promoters in the tammar *IGF2* region.

**Figure 3:**
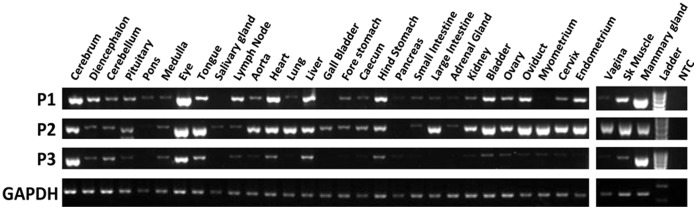
*IGF2* mRNA expression in the adult tammar. RT-PCR expression profile after 35 cycles of each *IGF2* mRNA transcript in a range of adult tammar tissues compared to *GAPDH*. The expression was strongest from P2, with expression of all three transcripts in the brain, eye, tongue, liver and mammary gland. No expression was detected in the No Template Control (NTC).

**Figure 4:**
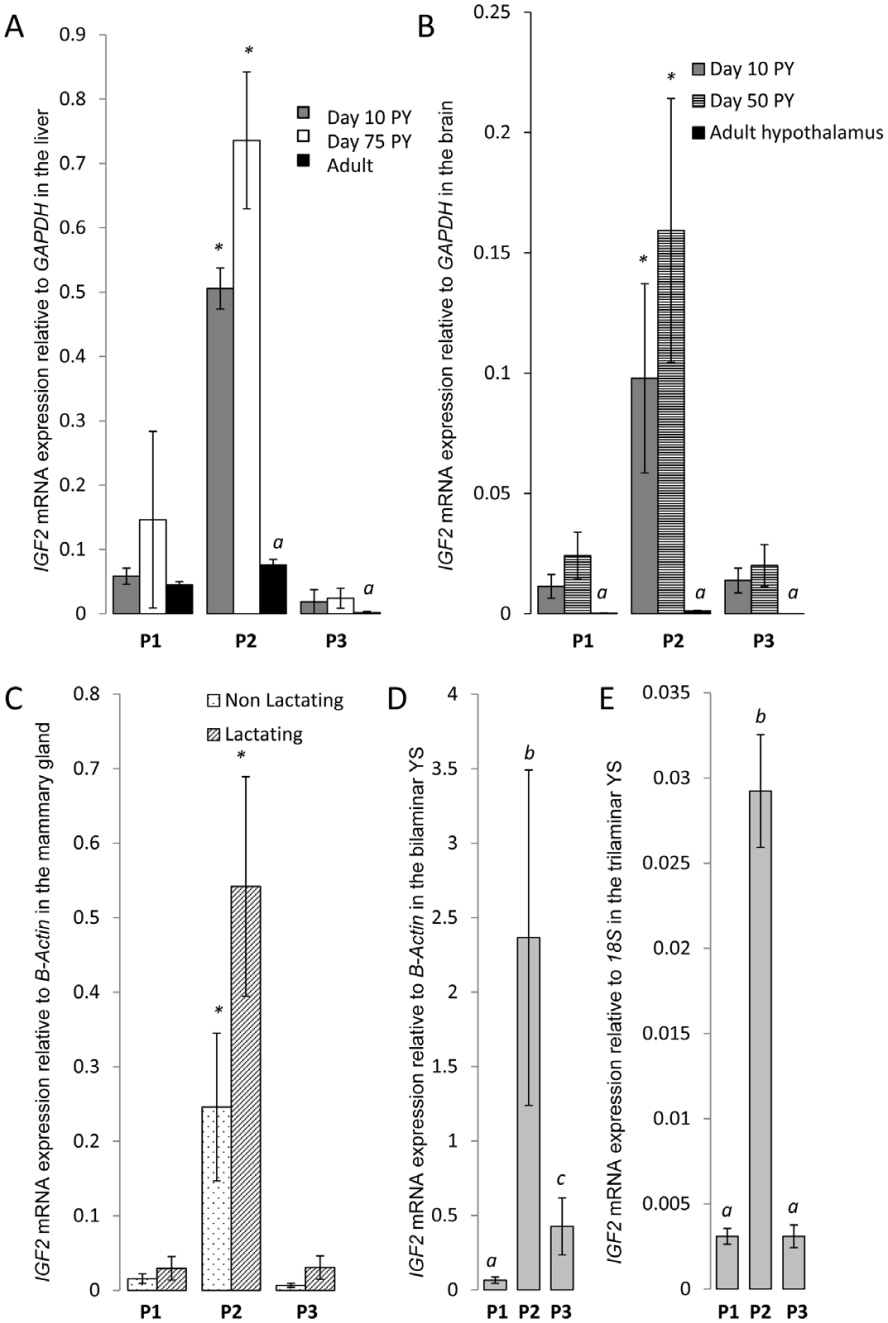
*IGF2* mRNA expression from each promoter (P1, P2 and P3) relative to a reference gene in various wallaby tissues. (**A**) *IGF2* mRNA expression relative to *GAPDH* in the Liver, (B) *IGF2* mRNA expression relative to *GAPDH* in the brain, (C) *IGF2* mRNA expression relative to *B-ACTIN* in the mammary gland, (D) *IGF2* mRNA expression relative to *B-ACTIN* in the bilaminar yolk sac and (E) *IGF2* mRNA expression relative to 18S in the trilaminar yolk Sac. (A, B) Expression from P2 in the pouch young (PY) is significantly higher than expression from P1 and P3 (* P<0.05) in the liver (A) and brain (B). Except for P1 in the liver, *IGF2* is expressed at a higher level in the pouch young then in the adult (*a*, P<0.01) in both the liver and the brain (if adult hypothalamus expression is representative of total adult brain expression). (C, D, E) P2-*IGF2* is also the predominantly expressed transcript in the mammary gland (* P<0.05) (C) and in the bilaminar (D) and trilaminar (E) placenta (a,b,c have significantly different means, P<0.01). There was no difference in the expression levels of any of the transcripts between non-lactating and lactating mammary glands (C).

## Materials and Methods

### Ethics Statement

All experiments and wild animal collection were approved by the University of Melbourne Animal Experimentation Ethics Committee and the animal handling and husbandry procedures were in accordance with the National Health and Medical Research Council of Australia (2004) guidelines. Animals were collected under approval from the South Australian Department of Environment and Natural Resources.

**Figure 5:**
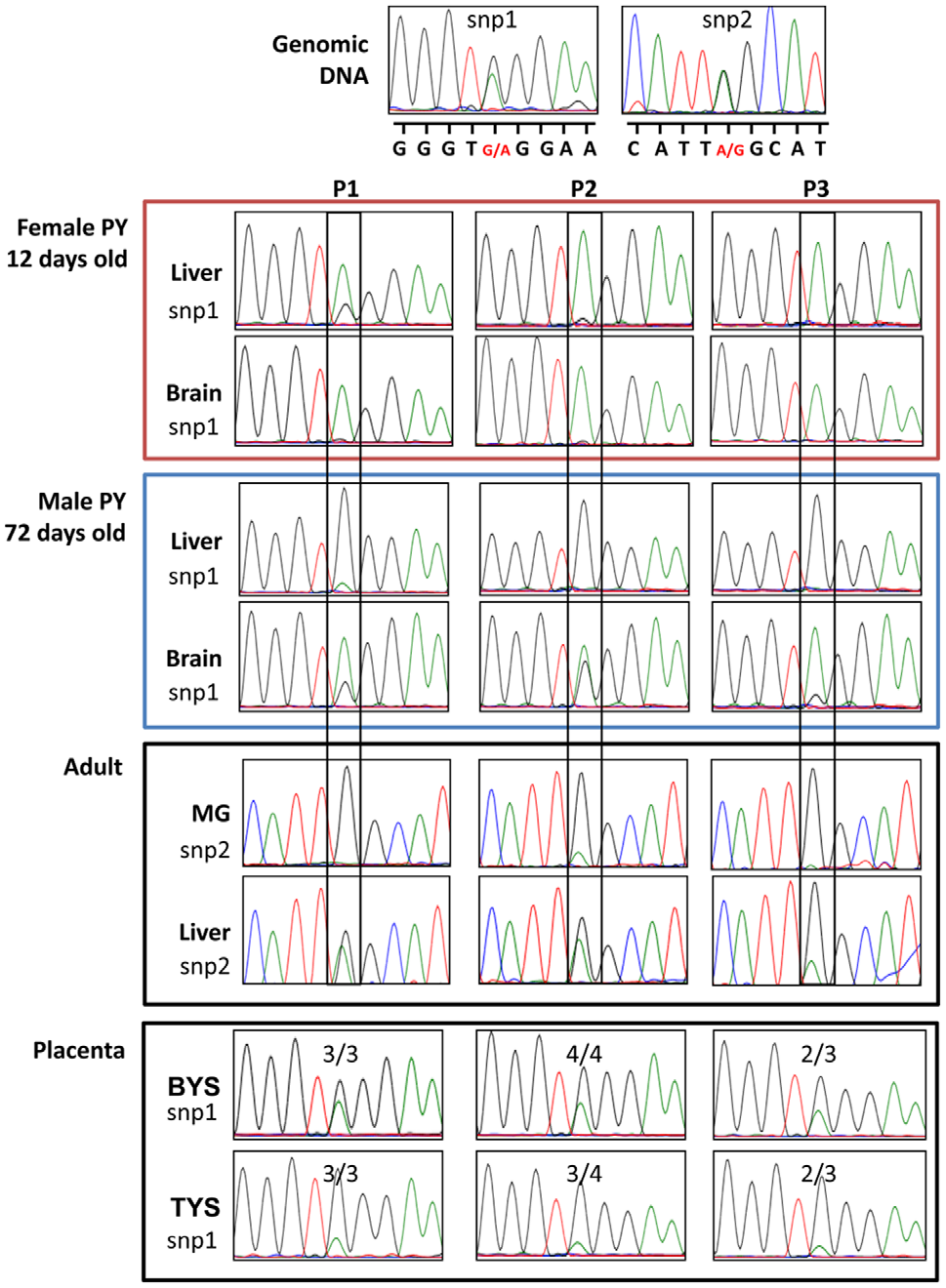
Allelic expression from the three promoters in various adult and pouch young tissues. Direct sequencing of two single nucleotide polymorphisms (snp1 and snp2) was used to determine the allelic expression of each of the *IGF2* transcripts in adult and pouch young (PY) tissues. In a 12 day old female pouch young monoallelic expression was detected from all 3 promoters in the brain and liver, except P1 in the liver was expressed from both alleles. In a 72 day old male pouch young, biased to biallelic expression was detected in the brain while the liver expression was similar to the day 12 pouch young. In the adult, both alleles were detected from all three TSS in two liver samples and monoallelic expression was detected in three mammary gland (MG) samples. Placental samples had biallelic expression in the bilaminar region (BYS) in all samples tested for P1 and P2 and 2 of the 3 samples tested for P3. Biased to monoalleleic expression was observed in the trilaminar region (TYS) in all samples tested for P1, 3 of the 4 samples for P2, and 2 of the 3 samples for P3.

### Animals

Tammar wallabies of Kangaroo Island, South Australia origin were held in our breeding colony in Melbourne. Pregnancy was initiated in females carrying an embryo in diapause by the removal of their pouch young (RPY) [37], [38]. Adult females carrying fetuses in the final third of gestation (day 19 to day 26 RPY of the 26.5 day pregnancy) or pouch young (day 0-day 350 post-partum) were either collected in the wild, or from our colony. The lactating mammary gland and adult and pouch young tissues were collected immediately after death and snap frozen in liquid nitrogen.

**Figure 6:**
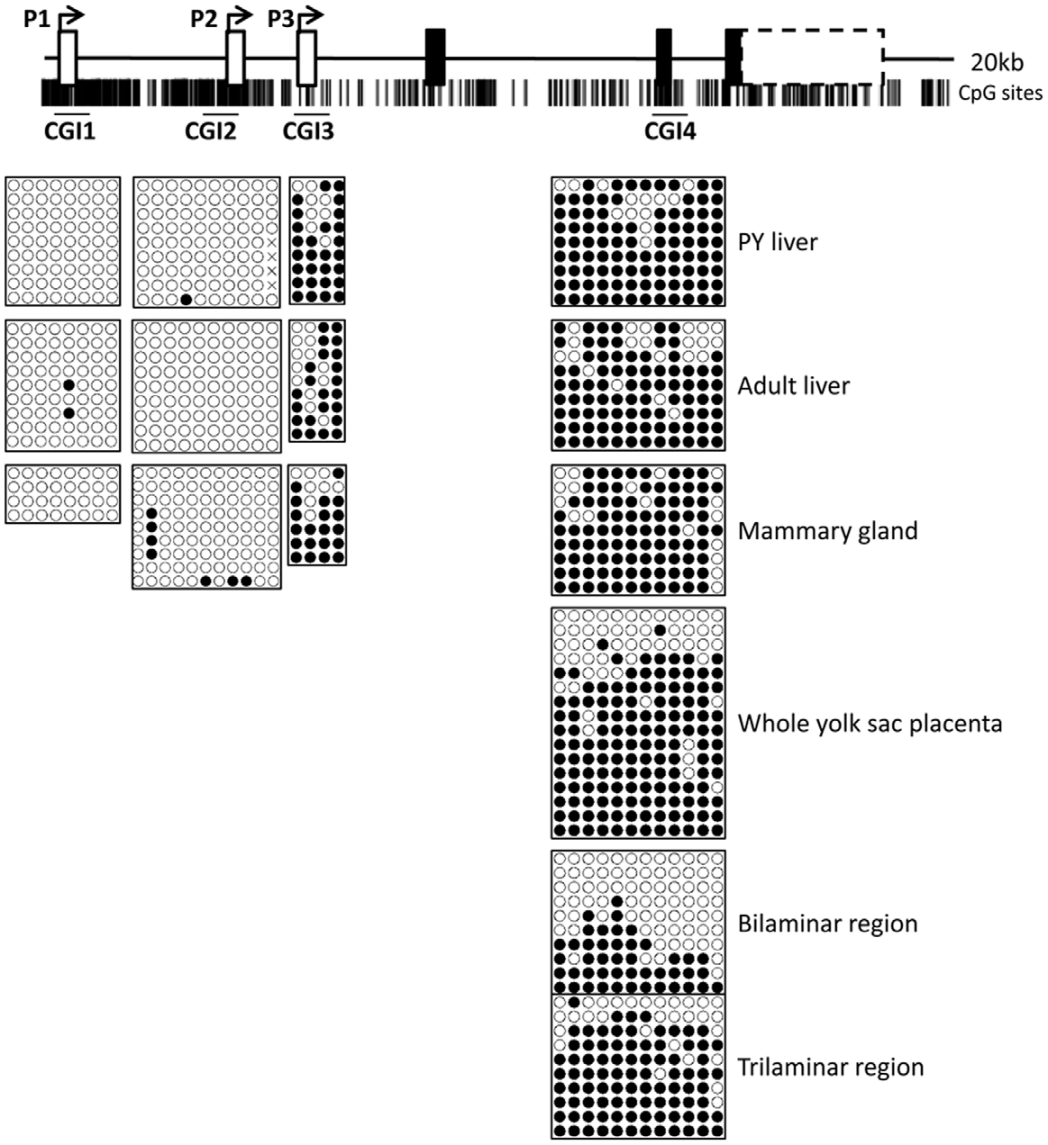
Methylation at the tammar *IGF2* transcription start sites. Location of CpG sites are represented by black bars below the schematic of the tammar *IGF2* gene. Bisulphite sequencing of the *IGF2* CpG islands (CGI) at each of the transcription start sites (CGI1, CGI2 and CGI3) and at exon 3 (CGI4) in the pouch young and adult liver, in the mammary gland, whole yolk sac placenta and in the bilaminar and trilaminar regions of the yolk sac placenta. Black circles represent a methylated CpG site and open circles represent an unmethylated CpG site. Each row represents the methylation pattern of a separate DNA fragment from the same sample. Only CGI3 and CGI4 were significantly methylated in all tissues. CGI4 appears to be differentially methylated in the placenta. However, sequence polymorphisms that might distinguish parental copies of CGI4 were not found among the available stocks of tammar placental samples.

### RNA and DNA Extraction and RT-PCR

Genomic DNA (gDNA) was extracted from approximately 20 mg of snap frozen tissue using a Wizard Genomic DNA Purification Kit (Promega). Total RNA was extracted from liver, placenta and brain tissues using Tri-Reagent (Ambion) as described by the manufacturer, and from mammary glands using RNeasy Lipid Tissue Mini Kit (QIAGEN), with a final elution of RNA in 50–80 µl of RNAsecure H_2_O (Ambion, Geneworks) in a dilution of 1/24. Total RNA was DNase treated (DNA-freeTM, Ambion) to remove contaminating DNA and tested by performing a housekeeping gene PCR. Total RNA quality was assessed by running samples on a gel (100%, 100 V for 30 min) and quantified with a nano-spectrometer (NanaDrop ND-1000 Spectrophotometer, NanoDrop Technologies Inc, Wilmington, DE, USA). cDNA was synthesised using Roche Transcriptor High Fidelity cDNA Synthesis Kit (Roche). Typically 4000 ng of total RNA was used in each cDNA synthesis reaction, with 1.1 µl of Oligo (dT)18 (50 µM), trilaminar yolk sac samples for qPCR analysis were amplified using random hexamer primers. All cDNA samples were diluted to 40 uL for a final concentration of 100 ug/ul. cDNA integrity was immediately assessed with *GAPDH* PCR.

All primers were designed using Primer3 (v. 0.4.0) [39] and synthesised by Sigma-Aldrich; (Table S1).

### 5′Race Amplification

To identify tammar *IGF2* transcription start sites (TSS) we performed 5′RACE, using both the 5′ RACE System for Rapid amplification of cDNA ends, Version 2.0, (Invitrogen) and SMARTer RACE cDNA Amplification Kit (Clontech). Liver and placental RNA was used. Gene specific primers were designed in the first translated exon (Table S1) and were used in conjunction with the provided Abridged Anchor Primer to amplify the 5′ end of the transcript.

PCR products were subcloned in to the pGEM-T Easy vector and transformed in to JM109 competent cells (Promega). Plasmids were purified using the Wizard Plus SV Minipreps DNA Purification System (Promega) and sequences were obtained according to standard methods using ABI3130xl capillary genetic analysers, with BDV3.1 terminators.

### 2.5 Quantitative RT-PCR Analysis

Quantitative real-time polymerase chain reaction (qPCR) was used to quantify the relative expression from each of the *IGF2* transcripts in the brain, liver, mammary gland and placenta. Each sample was extracted and prepared as described above. In the brain expression profile, 1 female and 3 male samples were used in the day 10 pouch young (PY) group and all 3 of the day 50 PY group were male. In the liver, 1 female and 3 male samples were used in the day 10 PY group and 2 female and 2 male samples were used in the day 75 PY group. All of the adult samples were female. As there were no obvious outliers in the data, we decided it was acceptable to use both sexes.

*IGF2* qPCR primers were designed so the reverse primer encompassed the intron exon boundary between exon 2 and 3 and was used with the transcript specific primers (Table S1).

PCRs were carried out in triplicates in 20-µl volumes consisting of FastStart Universal SYBR Green Master (Rox) (Roche), forward and reverse *IGF2* primers of an optimal concentration between 300–500 nM, and 1 µl cDNA template. Real-time PCR was carried out in an Stratagene Mx3000P™ Sequence Detector (integrated sciences) using the following conditions: 95°C for 10 min, followed by 45 cycles at 95°C for 15 sec and 61°C for 30 min and 72°C for 30 sec. A liver sample triplicate and a negative template triplicate were included on each plate as a calibrator and negative control respectively for each primer combination.

In addition to using the dissociation curve, PCR products from the first plate were run on a gel to confirm amplification of a single band and that the NTC wells were blank. The data was analysed in Microsoft Excel and R statistical package [40]. The amplification efficiency was calculated from the standard curve and Ct values corrected [41]. Paired t-tests were used in addition to one-way analysis of variance (ANOVA) and Tukey’s pair wise comparisons, to determine which results were significant. Means were considered different if P≤0.05.

### Imprinting and Expression Analysis

To determine the presence or absence of *IGF2* allelic expression sample specific genotypes were identified using PCR and direct sequencing of gDNA. Two single nucleotide polymorphisms [34] (Figure 1) were analysed by direct sequencing using common *IGF2* primers for gDNA and transcript specific primers for cDNA (Table S1) similar PCR conditions were used for each sample.

Each PCR reaction contained approximately 4 ng/µL of template and 0.2 µM of each primer. For imprinting analysis Ex Taq DNA polymerase, Hot-Start Version (TaKaRa) was used to optimise reaction efficiency and only 30–35 cycles of PCR were used to prevent saturation. For expression analysis GoTaq Green Master Mix (Promega) was used. PCR cycles consisted of 94 or 96°C for 2 min, followed by a maximum of 35 cycles of 30 sec at 94 or 96°C, 30 sec-1 min at 58–63°C, and 30 sec-1 min at 72°C, and a final extension at 72°C for 5 min.

Approximately 9 pouch young, 13 adults and 17 placental samples were screened for *IGF2* snps. Of these samples 2 adult livers, 3 mammary glands, 4 placentas and 2 pouch young brains and livers were analysed for *IGF2* imprinting. PCR products from cDNA and gDNA were resolved by gel electrophoreses and bands extracted (QIAquick Gel Extraction Kit, QIAGEN). The purified product was then sequenced. Sequences were assessed by eye using the FinchTV (v.1.3.1) DNA sequence chromatogram trace viewer software. The relative peak height for each allele indicates biallelic (equal peak heights) or imprinted (unequal peak heights) expression.

### Methylation Analysis

Using MethPrimer [42] several CpG sites were identified upstream of each of the non-coding exons. 1 ug of DNA was treated with a sodium bisulphite solution at 50°C for 4 hours before ethanol precipitating and eluting in 50 µl of TE. Approximately 20 ng of DNA was used as a template with 0.2 µM of each bisulphite (B/S) primer (Table S1) in a 25 µl reaction. To reduce the effects of PCR and cloning biases Takara Ex Taq DNA Polymerase (Hot-Start Version) was used to increase specificity, prevent background amplification, give higher yields and reduce sequence errors. PCR products generated with TaKaRa Ex Taq HS contain a mixture of 3'-A overhangs and blunt ends which allows >80% cloning efficiency in T-vectors. PCR cycles consisted of 96°C for 1 min, followed by 35 cycles of 30 sec at 96°C, 30 sec at 60°C, and 20 sec at 72°C, and a final extension at 72°C for 5 min. PCR products were cloned as described above and sequences were analysed using Quma quantification tool for methylation analysis [43].

## Results and Discussion

The regulation of the imprinted *IGF2* gene is highly complex in eutherian mammals. This study confirmed that marsupials also have complex regulation of *IGF2*, and identified three evolutionarily conserved promoters, each with three distinct non-coding exons that showed tissue specific imprinting and expression in the tammar wallaby.

### Identification of Three Transcription Start Sites in the Tammar Wallaby IGF2 Locus

To determine how many different transcription start sites (TSS) there are in the tammar *IGF2*, 5′ RACE was performed on mRNA derived from two tissues, liver and placenta. Three distinct bands were amplified from the liver and placental samples, representing three distinct TSS (Figure 1A). As additional faint bands were detected in placenta, 5′ RACE was repeated with a more sensitive kit (Clontech) (Figure 1B). Each band was cloned, sequenced and aligned to the genomic sequence. Three non coding exons were identified and named 1A, 1B and 1C each associated with a separate promoter P1, P2 and P3 respectively (Figure 1C). Three additional P2 TSS were detected in the placenta. Therefore, tammar *IGF2* may not have a liver specific promoter orthologous to human HuP1 or a placenta specific promoter orthologous to mouse P0.

### Evolutionary Conservation of Three IGF2 Alternate Promoters

At the mouse and rat P1 there are no TATA, CAT or GC boxes, but there are direct and inverted repeats of various length in the region upstream of the P1 non-coding exon [17], [44]. Similarly, no TATA-box, initiator element (Inr) or downstream promoter element (DPE) were located around tammar exon 1A TSS, although some repeat sequences were identified (Figure S1A). Tammar P2 had multiple transcription start sites, and had multiple CCATT and GC-box/Sp1-sites. Both a TATA-box and an Inr element was identified around the forth P2 TSS (Figure S1B) and these elements are predicted to act synergistically since the distance between the TATA-box and Inr is between 25–30 bp [45]. Tammar P3 is predicted to be a Inr-DPE core promoter as the DPE was located at precisely +28 to +32 relative to the A_+1_ nucleotide in the Inr motif (where A_+1_ is the transcription start site) [46]–[48] (Figure S1C)**.** A TATA-like sequence at -27 relative to the P3 A_+1_ nucleotide in the Inr motif, similar to the TATA-box observed at the mouse P3 [10], may have lost its consensus in marsupials after their divergence from the marsupial-eutherian ancestor. The conserved core promoter elements along with the arrangement of the three promoters suggests tammar promoters P1–P3 are orthologous to the human and mouse P1–P3.

Using 20 kb genomic sequences of *IGF2,* pairwise alignments using a 100 bp sliding window were performed in VISTA [49] between tammar (clone MEKBa-346C2∶42205-62204) and the base genome of mouse (ch7∶149834890-149854889) human (Ch11∶2149386-2169385), and opossum (clone XX-223O16 ch5.102112- 821130). Peaks (pink) represent areas with over 70% sequence conservation between tammar and opossum and over 55% between tammar and human and mouse (Figure 2). A minimum conservation width of 100 bp was used for alignments between marsupial species and 50 bp for alignments between eutherians and marsupials. Relative exon location was conserved between species with dense CpG sites around the three non-coding exons (Figure 2). There was a highly conserved peak between P1 and P2 in both eutherians and marsupials, suggesting that this was the ancestral arrangement of the three promoters and non-coding exons. As further evidence, the tammar alternate promoter derived non-coding exons were highly conserved in the opossum. However, there was limited sequence similarity between the tammar, mouse and human non-coding exons. The opossum, (family Didelphidae) and the tammar (family Macropodidae) are predicted to have diverged approximately 80 million years ago, whereas marsupials and eutherians last shared a common ancestor over 160 million years ago [31]. As these exons are non-coding, sequence changes in these exons will not change the function of the peptide. Therefore, it was expected that there would be far less sequence conservation between the marsupial and eutherian species than between the two marsupial species.

The opossum non-coding exon was highly conserved in the tammar genome and aligned to the tammar with 92% sequence identity approximately 300 bp upstream of the first tammar exon 1B TSS (Figure S2). This suggests that there may be another unidentified non-coding exon upstream of tammar exon 1B. There are no non-coding exons identified in monotreme *IGF2* [36]. Therefore, the additional promoters and exons must have arisen after the therian-monotreme divergence. The high level of sequence conservation of the tammar exons 1A, 1B and 1C in the opossum sequence suggests that these non-coding exons and promoters existed in the therian common ancestor before the eutherian - marsupial divergence and that the opossums may have lost the function of these promoters, or they are yet to be identified.

### Conserved Expression Patterns from Alternative IGF2 Promoters

IGF2 is synthesized in most tissues and its production declines rapidly in rodents soon after birth [19], [50]. In humans, circulating plasma levels of IGF2 remain at relatively high concentrations throughout life [51]. However, total *IGF2* transcription in the liver peaks shortly after birth [52]. Expression of *IGF2* in the tammar is similarly developmental stage-specific; expression is higher in the pouch young, during the time of rapid growth and nutrient transfer from the mother, compared to both fetal and adult stages [53]. Transcription from P1 and P2 appeared to be expressed in almost all of the adult tissues tested while expression from P3 was predominantly seen in the brain, mammary gland and eye (Figure 3). As in the human and in contrast to the mouse [14], [54], [55], transcription in the tammar was higher from P2 compared to P1 and P3 in the pouch young liver (P<0.005) and brain (P<0.05) (Figure 4A & 4B), in the adult mammary gland (P<0.05) and in the bilaminar and trilaminar regions of the placenta (P<0.01) (Figure 4C, 4D & 4E).

In the mouse, hepatic *IGF2* mRNA peaks approximately 10 days after birth [19]. In the tammar, hepatic *IGF2* expression peaks in the male pouch young between 70 and 100 days after birth and in females around 150 days [53]. As expected, expression in the tammar also varied with stage. In liver, P2 and P3 had significantly higher expression in the pouch young compared to the adult (P<0.01), but there was no significant difference in expression from P1 (Figure 4A). Expression of *IGF2* in liver was predominantly monoallelic from each of the 3 promoters in pouch young aged 12 and 72 days, and biallelic in both adults tested (Figure 5). As *H19* is not expressed in the adult liver, it is likely that the differentially methylated CTCF binding sites act as the imprinting control region in marsupials as it does in eutherians [33]. This epigenetic mark may be relaxed in the liver as the young grows, possibly by increased methylation on the maternal allele, allowing *H19* downstream enhancers to activate maternal *IGF2* promoters.

In the human overall *IGF2* mRNA expression is higher in the fetal brain compared to the adult [55], [56]. In tammar, expression from all three promoters in the adult hypothalamus was significantly lower than in the whole pouch young brain (P<0.01) (Figure 4B). *IGF2* RT-PCR amplification was detected in several regions within the brain including the cerebral cortex, cerebellum, diencephalon and pituitary (Figure 3), similar to the expression in the human brain [55], [57], [58]. In rodents, expression in the choroid plexus and leptomeniges are the only major sites of *Igf2* gene expression in the adult. In both humans and rodents, *IGF2* is biallelically expressed in a region-specific manner in the brain [1], [54], [55], [59]. As in the liver, imprinting in the tammar brain may be developmental stage-specific. Expression was monoallelic in the day 12 female PY but in the day 72 male there was expression from both alleles (Figure 5). This could suggest that the imprinting mark is relaxed with age or, as in eutherians, some regions in the brain may maintain monoallelic expression while other regions, such as the choroid plexus, may express *IGF2* biallelically. In the tammar pouch young liver, *IGF2* expression levels vary between males and females with a significant difference at day 70 post partum [53]. Thus, another possibility, although less likely, is that *IGF2* imprinting in the brain is sex specific, as observed at the *Interleukin 18* locus in the mouse [60].

There was no significant variation in the expression of the three different transcripts in the mammary glands between early and mid lactation (Figure 4C). Both P1 and P3 were monoallelically expressed while P2 showed some expression from the silenced allele (Figure 5). All three mammary glands tested produced the same result. If imprinting is maintained in organs that influence maternal nutrient transfer, monoallelic expression of *IGF2* may be a selective advantage in the adult mammary gland [35], but not in the liver.

### Imprinting in the Placenta

The pattern of expression from the three promoters appeared to be conserved in both the trilaminar and bilaminar regions of the chorio-vitelline placenta with the highest expression from P2 compared to both P1 and P3. The imprint status was analysed in 4 different placentas, aged between days 21–26 of pregnancy. Interestingly, the expression of the three *IGF2* promoters in the vascular trilaminar region was predominantly from one allele while both alleles were expressed in the bilaminar yolk sac placenta (Figure 5).

Expression of *IGF2* is significantly higher in the trilaminar yolk sac compared to the bilaminar [61]. The avascular bilaminar yolk sac serves primarily in the uptake of nutrients from the uterine secretions, while the vascular trilaminar yolk sac is important for respiration [62], [63]. *IGF2* has been implicated in several aspects of placental development, including blood vessel formation [64] and gaseous transfer [20], [21], [65]. Therefore, monoallelic expression of *IGF2* may be selectively advantageous for vascular growth specifically in the vascular trilaminar placenta.

The tammar promoters P1–P3 were imprinted in the placenta and pouch young with selectively maintained imprinted expression in adult tissues, including the mammary gland. In mouse and human, these three promoters are mainly expressed in the fetus and placenta with expression levels dropping off, and in some cases becoming biallelically expressed, in the adult [1], [14], [15]. In the tammar, the duration of development of the young in the pouch is equivalent to fetal stages of eutherians, so the tammar *IGF2* promoters would be expected to have similar expression patterns and imprint patterns in the pouch young as they do in the eutherian fetus. However, expression was maintained in the adult tammar, albeit at lower levels, suggesting that the regulation of these promoters is more similar to that in humans than in the rodents.

### DNA Methylation in the Promoter Regions of Tammar IGF2

Promoter specific methylation in adult humans increases with age as expression levels decrease [16], [29]. In the tammar, CpG islands (CGI) were located around each of the transcription start sites as well as across exon 3 (CGI4, the location of eutherian DMR2) (Figure 6). While it is still unknown whether DNA methylation at promoters is the primary cause or consequence of gene silencing, the expression of tammar *P2-IGF2* significantly decreased in the adult liver in absence of increased TSS methylation (Figure 6). Therefore, it is unlikely that methylation at the P2 TSS controls the expression levels in the liver. The presence of multiple Sp1 sites (Figure S1) may explain the lack of methylation observed at this promoter. Deletion or mutation of mouse Spl sites within the unmethylated *Aprt* CpG island causes de novo methylation [66], [67]. However, the region between P1 and P2 is very CpG rich (Figure 6) so methylation further upstream of the P2 may regulate expression. The P3 TSS was highly methylated without any obvious allelic bias in young or adult liver and mammary gland (Figure 6). As P3-*IGF2* transcript number is usually lower in the adult tissues relative to P1- and P2- *IGF2* expression, methylation may function to regulate the expression levels at this promoter.

In the mouse, methylation at DMR1 and DMR2 on the expressed paternal allele correlates significantly with the level of overall *IGF2* expression [19]. Differential methylation was not detected at CGI4, in either the mammary gland or liver and a similar level of methylation was detected at the P3 TSS (Figure 6). There was, however, a possible DMR in the placenta at the CGI4, with a higher level of methylation in the trilmainar region compared to the bilaminar region (Figure 6). This suggests that methylation at this site may regulate either the expression levels and/or the imprint status of *IGF2* in the placenta. Sequence polymorphisms that might distinguish parental copies of CGI4 were not found among the available samples. However, the presence of hypo- and hyper- methylated alleles is indicative of a DMR. In eutherians, the DMR2 is predicted to be an enhancer [27], so in the tammar this region may also be a tissue or cell specific activation site.

### Conclusion

The expression and imprinting of *IGF2* is controlled in a developmental- and tissue-specific manner in both eutherians and marsupials. One differentially methylated TSS has previously been reported in the opossum [13]. While not differentially methylated, at least three promoters appear to be functional in the tammar. The conserved core-promoter elements in addition to the conserved sequence between P1 and P2 in both eutherians and marsupials suggest these promoters were present in the eutherian-marsupial ancestor. The expression pattern of these three transcripts mirrors that which is seen in humans, with predominant expression from the P2 promoter and continued expression into adulthood, despite their differences in reproductive strategies. Interestingly, unlike the human and opossum, tammar *IGF2* lacks promoter specific DMRs, similar to that which has been reported in the mouse [29], [30]. While marsupial *IGF2* imprinting is likely to be regulated by the *H19* ICR, as in eutherians [33], the differences in the promoter methylation patterns between human, mouse, opossum and tammar demonstrate divergence in some epigenetic mechanisms. However, the existence of three orthologous promoters and a putative DMR across exon 3 in the tammar, human and mouse provides evidence of a conserved origin of imprinting mechanisms for this gene between marsupials and eutherians.

## Supporting Information

Figure S1 **Nucleotide sequence of the three tammar** ***IGF2*** **gene promoters.** The DNA sequences of *IGF2* promoters P1, P2 and P3 are illustrated in A, B, and C, respectively. Transcription start sites (TSSs) identified by 5′RACE are indicated by the arrows. ggaggag repeats are in boxes, CTCCCAAG repeats are highlighted in yellow and TGTCCC repeats are highlighted in green. TATA-boxs (TATAWAAR) are emboldened and red, CCATT and GC-box/Sp1-sites are underlined, Inr elements (YYANWYY) are emboldened and blue and DPE (RGWYV) sequences are emboldened and green. Exon sequences are in capitals. P1 is characterised by multiple repeat sequences. P2 has multiple TSSs and a high number of CCATT and GC-box/Sp1-sites. A TATA-Inr core promoter was identified around the fourth TSS. P3 is an Inr-DPE core promoter. Degenerate nucleotides represented using IUPAC codes.
(PDF)

Figure S2 **Alignment of tammar exon 1B region with opossum exon 1 region.** Opossum non-coding exon = 1–420 Tammar exon 1B = 725–1104. Alignments were performed using ClustalW and were highlighted using BOXshade 3.31.
(PDF)

Table S1 **Primers used in this study.**
(PDF)
