# Supplementary material for: Promoter-Specific Expression and Imprint Status of Marsupial IGF2
Source: PLoS One. 2012 Jul 25;7(7):e41690. doi: 10.1371/journal.pone.0041690 (PMC3405008; doi:10.1371/journal.pone.0041690)
Supplement: Figure S1 — Nucleotide sequence of the three tammar IGF2 gene promoters. The DNA sequences of IGF2 promoters P1, P2 and P3 are illustrated in A, B, and C, respectively. Transcription start sites (TSSs) identified by 5′RACE are indicated by the arrows. ggaggag repeats are in boxes, CTCCCAAG repeats are highlighted in yellow and TGTCCC repeats are highlighted in green. TATA-boxs (TATAWAAR) are emboldened and red, CCATT and GC-box/Sp1-sites are underlined, Inr elements (YYANWYY) are emboldened and blue and DPE (RGWYV) sequences are emboldened and green. Exon sequences are in capitals. P1 is characterised by multiple repeat sequences. P2 has multiple TSSs and a high number of CCATT and GC-box/Sp1-sites. A TATA-Inr core promoter was identified around the fourth TSS. P3 is an Inr-DPE core promoter. Degenerate nucleotides represented using IUPAC codes. (PDF) [file pone.0041690.s001.pdf]

A- P1

Ctttgtcccttttttgtcgattggagacccaaggactaactgtgcctggttgaatatttgcctttaaagcaaagcctggactttgcccctctgccatttcaggtt
tcatttcctcagaaaggctttttttgtggaggagaaagaaggcgctccgaggtacccctacccgttgccacccctggtccgcccgcgcgcgcttctctc
cccagatcttgtattttctgctgggataaacctgaactcgtccccagcctcgcttctctttttaaaggtcaagacagagtgaccccggacgcttggggtgc
ctgcagcctgggagttcctggcctcccaggccgctccccccctccaccccggttcactgagcacccaggactgcctggttttccgtatggaaggcgcc
ggctttgaggagacgcgtattgtcttcctcccaaggcggcagccccgctcccaagcgcacgcctcacggctcccttgtccgttgtccccgtcctctgtc
cccgcgccgccccctacaattcacacgcacgcacgc*acacaca*t*acacacacacacacacacacacacacacacacaca*tttcgctgttta
tttgccacggaggagggataacgaaggagcAATTAAGCGTTCAAAGCCTCGCTAGACAGCACACCGCCGCAGCCT
TCGCAAAATCTGG...3'

B- P2

cgggaattcgcagcgtcggctcaccatcttcgcgtcgccgccgcctttccatcccatccgacgacgtgggcgctgcccccgcctcactttccatcctgc
aggcgcccggcgcccctccgtcaatcaacgtccttccccccctcccctcccttcccccctccttttttttttttttttttgctgctaattggggcctctgtgcgga
acgccaaactgccttttccttaactacctcagctttcatcccattccacctcccttaaagggaaaaggcaggttccgcctccagaggtctcatctccttcc
ccgccctcagtccgccccacaggatcccccaaattgcagcccattctctctgtagcctgactccggaggcggaggactcaggcccgcggggtgcct
cgcagaaggcgtccccccgacgtgggaggcagtttggtccccttcccttgcccttagcttaaatcgctctgtcttccctccccaccctcgcacccatcgc
cctttctccccgttctccctcctaagtacccccccccggtcctccccgccctgcccggcccgcccctgtctgcccgcaccctcggccctcgccctctct
ctctgtctcctgttctctgcCCCCTCTGCCCCCTCTGGCTTCGCTCGCTCGCTGCACGCCGCCCTCGCTCACTC
TCTGTCTGTCGCCTGTGCTCTATCTTTCTCACTGACTGTCTTCTGCTACTTCTGTCCACTGTTCTACAA
AACCCTCTTCACTATCTCCCCAAACTTATCCAAAAACAACGAGTCAACCCGCCCCGCCCCAAAGCCC
GTCCACTTGAATAAAAAAAAAAAAAA**AGACA**AAAAAACCTAACTACGCCCCCAA**TATAAAAG**CCAGAA
CCAGCCTGACCCGAATCCAAAA**TTAGACC**AATGATGAGATTTCGTCAGCGCGGGAACACGGCAAGA
GGAGTCGCTTCAGGGTCGGCGGACGCGAGTGTCAGCACCCTACAACAGTCCCGCAGCAAGGAGGT
GGGCAGTAGCAGTGGCAGCTTTGA...3'

C- P3

aacctgaatttgaagggaggtctgttgcctgctaactttataaaataccctcagcttccccaaggatgctactcattggcataggaaaaccaaggtcca
gaacagctaagccaaatgttgggggggaggaaggggcagccacattactcttggggtatgccggtagacagtgccaatatctgaaagagttcatga
aatctgtgtggtatctctgggtaggggaaagtttttttgcccaggaatctgacaggatagggaaaggtgccttataggctgagtccctagtgtttgttagtta
ggatccattaggcagcacagacccagatggggcagggttatactgatcttggatccctgaagccgtgggtctctgggctagggctgtagggggtggt
gccactttggcaaatccttgaggacccaggtaatggtgatgtaatctgggaattgttccagagtgcctcttagggaccctgatatcattggctgagctga
aggatgggggattgggtgagctgaagaagtaggaggagattagggagggacaagcaggggcgtggttatggactgggttggactttgggggctg
ggggctac**ataaatgt**gtgagcacagatcagctc**tcAGTTT**CGGAGATCACTGAGAGCGGAGC**AGTCA**CCCTCCAAG
CCTTCTGCTGGGCACTGGTGGAGAGACAGAAGACAGCCTCACTCTTGGAAAAGCTCACG...3'

Figure S1 Nucleotide sequence of the three tammar *IGF2* gene promoters. The DNA sequences of *IGF2* promoters P1, P2 and P3 are illustrated in A, B, and C, respectively. Transcription start sites (TSSs) identified by 5´RACE are indicated by the arrows. ggaggag repeats are in boxes, CTCCCAAG repeats are highlighted in yellow and TGTCCC repeats are highlighted in green. TATA-boxs (TATAWAAR) are emboldened and red, CCATT and GC-box/Sp1-sites are underlined, Inr elements (YYANWYY) are emboldened and blue and DPE (RGWYV) sequences are emboldened and green. Exon sequences are in capitals. P1 is characterised by multiple repeat sequences. P2 has multiple TSSs and a high number of CCATT and GC-box/Sp1-sites. A TATA-Inr core promoter was identified around the fourth TSS. P3 is an Inr-DPE core promoter. Degenerate nucleotides represented using IUPAC codes.
