# Supplementary material for: Promoter-Specific Expression and Imprint Status of Marsupial IGF2
Source: PLoS One. 2012 Jul 25;7(7):e41690. doi: 10.1371/journal.pone.0041690 (PMC3405008; doi:10.1371/journal.pone.0041690)
Supplement: Figure S2 — Alignment of tammar exon 1B region with opossum exon 1 region. Opossum non-coding exon = 1–420 Tammar exon 1B = 725–1104. Alignments were performed using ClustalW and were highlighted using BOXshade 3.31. (PDF) [file pone.0041690.s002.pdf]

```
opossum      1 AGTGGTGACATCCAGCGATCGGAGCGAGTTCTCCCAAACCGTTATAAAAGGAGAGCTGGA
tammar       1 AGTAGTGACATCCAGCGATCCGAGCGGGTTCTCCCAAACCGTTATAAAAGGACCGCAGGA
consensus    1 *** **************** ***** *************************  ** ***

opossum     61 GGGCGGATCTGTCTGTCTCCTCCTAGACGGAGCAGGTAGTCGGGGACTCGCAGCGTCGCC
tammar      61 GGACGGATCTGTCTGTCTCCTCCTAGACGGAGCAGGTAGTCGGGAATTCGCAGCGTCGGC
consensus   61 ** ***************************************** * *********** *

opossum    121 TCACCATCTTCGCGTCGCCGCCGCCGCCGCCGCCGCCGCCTTCCCATCCCATCCGACGAC
tammar     121 TCACCATCTTCGCGTCGCCGCCGCC---------------TTTCCATCCCATCCGACGAC
consensus  121 *************************               ** *****************

opossum    181 CTGGGCGCTGCCCCCGCCTCACTCACTTTCCATCTCGCAGGCGGCCGGCGCCCCTCCGTC
tammar     166 GTGGGCGCTGCCCCCGCCTCA----CTTTCCATCCTGCAGGCGCCCGGCGCCCCTCCGTC
consensus  181  ********************    *********  ******* ****************

opossum    241 AATCAGCGTCCTTCCCCC-TCCCCTTCCCCTTCCCCCTCCTTTTTTT------------G
tammar     222 AATCAACGTCCTTCCCCCCTCCCCCTCCCCTTCCCCCTCCTTTTTTTTTTTTTTTTTTTG
consensus  241 ***** ************ ***** **********************            *

opossum    288 CTGCTAATTGGGGCCTCTGCGAGGAGCACTACGCTGCCTTTGCCTTCACTACCTCAGCTT
tammar     282 CTGCTAATTGGGGCCTCTGTGCGGAACGCCAAACTGCCTTTTCCTTAACTACCTCAGCTT
consensus  301 ******************* * *** * * *  ******** **** *************

opossum    348 TCATCCCAGTCCACCGCCCTTACAGGCAAA-GGCAGGTTCCGCCTCCGGAGGTCCCATCT
tammar     342 TCATCCCATTCCACCTCCCTTAAAGGGAAAAGGCAGGTTCCGCCTCCAGAGGTCTCATCT
consensus  361 ******** ****** ****** *** *** **************** ****** *****

opossum    407 CCCTCCCCGCCCTCAG----------AGAATCCCCCAAATTGCAGTCCACCATCTCTGCA
tammar     402 CCTTCCCCGCCCTCAGTCCGCCCCACAGGATCCCCCAAATTGCAGCCCATTCTCTCTGTA
consensus  421 ** *************          ** **************** ***   ****** *

opossum    457 GCCTGACTCCGGGCGGCGGCGGCAGCGGCGGCAGCGGAGGAGGAGGAGGAGGAGGAGGAG
tammar     462 GCCTGACTCCGGA-GGCGGAGGACTCAG-GCCCGCGGGG---------------------
consensus  481 ************  ***** **   * * * * **** *

opossum    517 GAGGAGGAGGAGGAGGAGGAGGAGGAGGAGGAGGAGGAGGAGGAGGAGGAGGAGGAGGAG
tammar     499 ------------------------------------------------------------
consensus  541

opossum    577 GAGGAGGAGGAGGAGGAGGAGGAGGAGGAGGAGGAGGAGGAGGACTCCGGCTCTCGGGGA
tammar     499 -----------------------------------------------TGCCTCGCAGAAG
consensus  601                                                 * *** * *

opossum    637 GT---CCCCCGACGTGGGAGGCGGTTTGGTCCCCTGCCCTTGCCCTTAGCTTCAGTTGCT
tammar     512 GCGTCCCCCCGACGTGGGAGGCAGTTTGGTCCCCTTCCCTTGCCCTTAGCTTAAATCGCT
consensus  661 *    ***************** ************ **************** * * ***

opossum    694 --GTCTTCCCGCCCT-CCCTCGC-CCCGTCCCCCTTCGGGCCCTTTCTCCCTCCTGAA--
tammar     572 CTGTCTTCCCTCCCCACCCTCGCACCCATCGCCCTTTCTCCCCGTTCTCCCTCCTAAGTA
consensus  721   ******** ***  ******* ***.** *****    *** *********** *

opossum    748 --CTCCCCCCGATCCTCCCCGCCCTGCTCA-CCCGCCC----------------TCGGCC
tammar     632 CCCCCCCCCCGGTCCTCCCCGCCCTGCCCGGCCCGCCCCTGTCTGCCCGCACCCTCGGCC
consensus  781   * ******* *************** *  *******                ******

opossum    789 CTCGG--------TCTGTCTCCTTT----------CTCTGCCCCCTCTGGCTTCG-----
tammar     692 CTCGCCCTCTCTCTCTGTCTCCTGTTCTCTGCCCCCTCTGCCCCCTCTGGCTTCGCTCGC
consensus  841 ****         ********** *          ********************

opossum    826 ---------GCCGCCCGCG---GCTCTCTGTC----GCCCTTGCGCTGTCCTTGTC--TG
tammar     752 TCGCTGCACGCCGCCCTCGCTCACTCTCTGTCTGTCGCCTGTGCTCTATCTTTCTCACTG
consensus  901          ******* **    *********    ***  *** ** ** ** **  **

opossum    868 CCTGTCT------------GTCCGCGGTTCTGCCGAGCCCTCTCCACTCTCTCCCCAA-C
tammar     812 ACTGTCTTCTGCTACTTCTGTCCACTGTTCTACAAAACCCTCTTCACTATCTCCCCAAAC
consensus  961  ******            **** * ***** *  * ****** **** ********* *

opossum    915 GCGGCCCAAAACAACAAGTCAGCCCGCCCCGCCCCAGAGCCCGCCTGCCCGTCCG-----
tammar     872 TTATCCAAAAACAACGAGTCAACCCGCCCCGCCCCAAAGCCCGTCCACTTGAATAAAAAA
consensus 1021     ** ******** ***** ************** ****** *  *  *

opossum    970 ----------CCCGTCCGCCCGTCCGCTCGCGGGACTAGAGAAGCCCCAAAGCCCCGCTA
tammar     932 AAAAAAAAGACAAAAAAACCTAACTACGCCCCCAA-TATAAAGCC--AGAACC-----A
consensus 1081           *       **   *  * * *   * ** * *****  * * **     *

opossum   1020 GCCTG-CCCGAATCCCCGATTGGCCCGGGATGGGATTGGGGCAGCGAGGGAACATGGCG
tammar     984 GCCTGACCCGAATCCAAAATTAGACCAATGATGAGATTTCGTCAGCGCGGGAACACGGCA
consensus 1141 ***** *********   *** * **   **** ****  * ***** ******* ***
```

```
opossum   1079 AGAGGAGTCACTTCTGGGTCTGCGGACGCGCGCGTCGGCACCCTCCAACAGTCCCGCAGC
tammar    1044 AGAGGAGTCGCTTCAGGGTCGGCGGACGCGAGTGTCAGCACCCTACAACAGTCCCGCAGC
consensus 1201 ********* **** ***** ********* * *** ******* ***************

opossum   1139 AAGGAGGTGGGGAGGAGCAGGAGCAGCTCCGAG
tammar    1104 AAGGAGGTGGGCAGTAGCAGTGGCAGCTTTGA-
consensus 1261 *********** ** *****  ******  **
```

Supplementary Figure S2. Alignment of tammar exon 1B region with opossum exon 1 region. Opossum non-coding exon = 1-420 Tammar exon 1B = 725-1104. Alignments were performed using ClustalW and were highlighted using BOXshade 3.31.
