# Supplementary material for: Promoter-Specific Expression and Imprint Status of Marsupial IGF2
Source: PLoS One. 2012 Jul 25;7(7):e41690. doi: 10.1371/journal.pone.0041690 (PMC3405008; doi:10.1371/journal.pone.0041690)
Supplement: Table S1 — Primers used in this study. (PDF) [file pone.0041690.s003.pdf]

Supplementary Table S1. Primers used in this study.

Experiment/Gene	Primer	Primer sequence (5′ to 3′)
5′RACE	GSP1-s275	GCAGGGGTAGCACAGTAGGTCTC
	GSP2-s117	GTCTACCAGTTCCCCACCACAAAG
	GSP2-s 50	GCCAAAAAGGTGAGTGATAACAGGA
Imprint and expression	gDNAFw	GACTCCACTTTCTTCCTTCCCTTT
	cDNA-1A-Fw	CTTCGCAAAATCTGGATGGGTTT
	cDNA-1B-Fw	AGTGGCAGCTTTGAATGGGTTT
	cDNA-1C-Fw	CTCACTCTTGGAAAAGCTCACGAT
	Seq Fw	GAGAGGGCCTCGCTACCAAC
	SNP1-Rv	AAAGCATGGCAGCCCACACT
	SNP1-3Rv	CTCTTCTGCCGAAGGATGAC
	qPCR-Rv	TGCCAGGAAGACTGAAATAGAAGC
	gDNA-CGI4-Fw	GTGGCCAAGAGCCAGGCAAG
	gDNA-CGI4-Rv	AGGGCGAGGACAGACTGGAAG
Bisilphite sequencing	P1-BS- Fw	GGGAGTTTTTGGTTTTTTAGGT
	P1-BS- Rv	AAAAAAAACATACCCCAAATTTTAC
	P2-BS1- Fw	TTTTTGTTTATTGTTTTATAAAATTTTT
	P2-BS1- Rv	CACTACTACTACCCACCTCCTTACTAC
	P2-BS2- Fw	TTTAAAATTAGATTAATGATGAGATTT
	P2-BS2- Rv	CTACTACTACCCACCTCCTTACTAC
	P3-BS- Fw	TGGGGTAGGGTTATATTGATTTTGGAT
	P3-BS- Rv	TCTATCTCTCCACCAATACCCAACAAA
	CGI4-BS -Fw	AGTGAGGGAGAAGAGGGTTTTATTA
	CGI4-BS -Rv	AACCTCTTACCTCCCAAATTAACTC
B-ACTIN	Fw	TTGCTGACAGGATGCAGAAG
	Rv	AAAGCCATGCCAATCTCATC
18S	Fw	CGGCTACCACATCCAAGGAA
	Rv	GCTGGAATTACCGCGGCT
GAPDH	qPCRFw	TCCCAATGTATCTGTTGTGGATCTG
	qPCRRv	AACCATACTCATTGTCATACCAAGAAAT
	Fw	CCTACTCCCAATGTATCTGTTGTGG
	Rv	GGTGGAACTCCTTTTTTGACTGG
